# Association of artificial intelligence-based immunoscore with the efficacy of chemoimmunotherapy in patients with advanced non-squamous non-small cell lung cancer: a multicentre retrospective study

**DOI:** 10.3389/fimmu.2024.1485703

**Published:** 2024-11-06

**Authors:** Jiaqing Liu, Dongchen Sun, Shuoyu Xu, Jiayi Shen, Wenjuan Ma, Huaqiang Zhou, Yuxiang Ma, Yaxiong Zhang, Wenfeng Fang, Yuanyuan Zhao, Shaodong Hong, Jianhua Zhan, Xue Hou, Hongyun Zhao, Yan Huang, Bingdou He, Yunpeng Yang, Li Zhang

**Affiliations:** ^1^ State Key Laboratory of Oncology in South China, Guangzhou, China; ^2^ Collaborative Innovation Center for Cancer Medicine, Guangzhou, China; ^3^ Guangdong Provincial Clinical Research Center for Cancer, Guangzhou, China; ^4^ Department of Intensive Care Unit, Sun Yat-sen University Cancer Center, Guangzhou, China; ^5^ Department of Medical Oncology, Sun Yat-sen University Cancer Center, Guangzhou, China; ^6^ Bio-totem Pte Ltd, Suzhou, China; ^7^ Department of Anesthesiology, Sun Yat-sen University Cancer Center, Guangzhou, China

**Keywords:** NSCLC, immunotherapy, artificial intelligence, pathology, immunoscore

## Abstract

**Purpose:**

Currently, chemoimmunotherapy is effective only in a subset of patients with advanced non-squamous non-small cell lung cancer. Robust biomarkers for predicting the efficacy of chemoimmunotherapy would be useful to identify patients who would benefit from chemoimmunotherapy. The primary objective of our study was to develop an artificial intelligence-based immunoscore and to evaluate the value of patho-immunoscore in predicting clinical outcomes in patients with advanced non-squamous non-small cell lung cancer (NSCLC).

**Methods:**

We have developed an artificial intelligence–powered immunoscore analyzer based on 1,333 whole-slide images from TCGA-LUAD. The predictive efficacy of the model was further validated in the CPTAC-LUAD cohort and the biomarker cohort of the ORIENT-11 study, a randomized, double-blind, phase 3 study. Finally, the clinical significance of the patho-immunoscore was evaluated using the ORIENT-11 study cohort.

**Results:**

Our immunoscore analyzer achieved good accuracy in all the three cohort mentioned above (TCGA-LUAD, mean AUC: 0.783; ORIENT-11 cohort, AUC: 0.741; CPTAC-LUAD cohort, AUC: 0.769). In the 259 patients treated with chemoimmunotherapy, those with high patho-immunoscore (n = 146) showed significantly longer median progression-free survival than those with low patho-immunoscore (n = 113) (13.8 months vs 7.13 months, hazard ratio [HR]: 0.53, 95% confidence interval [CI]: 0.38 – 0.73; *p* < 0.001). In contrast, no significant difference was observed in patients who were treated with chemotherapy only (5.07 months vs 5.07 months, HR: 1.04, 95% CI: 0.71 – 1.54; *p* = 0.83). Similar trends were observed in overall survival.

**Conclusion:**

Our study indicates that AI-powered immunoscore applied on LUAD digital slides can serve as a biomarker for survival outcomes in patients with advanced non-squamous NSCLC who received chemoimmunotherapy. This methodology could be applied to other cancers and facilitate cancer immunotherapy.

## Introduction

Lung cancer, stands as the most prevalent cancer globally, responsible for approximately one in eight cancers globally (1). Despite significant advancements in lung cancer treatment, the prognosis for non-small cell lung cancer (NSCLC) patients remains poor, because most cases are diagnosed at an advanced stage (2, 3). Immune checkpoint inhibitors (ICIs) have changed the paradigm of NSCLC management, proven to be superior in survival outcomes compared with chemotherapy in patients diagnosed with advanced non-squamous NSCLC (4–6). Our previous study has also shown that chemo-immunotherapy could significantly improve the survival of non-squamous NSCLC in first-line therapy, with tolerable toxicity (7, 8). Although the response rate of chemo-immunotherapy in first-line therapy for non-squamous NSCLC is encouraging, the proportion of patients who could gain long-term survival benefits is still limiting; therefore more accurate predictive biomarkers are required (8, 9). Although biomarkers like PD-L1 expression and tumor mutation burden (TMB) have been shown to help predict immunotherapy efficacy, their effectiveness is also limited by the overlap between responders and non-responders, underscoring the necessity for more reliable biomarkers to guide clinical decisions (10–12).

In recent years, artificial intelligence (AI), especially machine learning (ML) and deep learning (DL) approaches, has shown considerable promise in improving the prediction of immunotherapy outcomes in NSCLC (13). AI-driven analysis of imaging data has made it possible to identify predictive biomarkers that correlate with immunotherapy responses. For instance, AI systems could extract subtle imaging features from noninvasive radiomic scans and quantify them by correlating imaging data with PD-L1, thus allowing for accurate prediction of PD-L1 status (14). Unlike traditional biopsies, AI-driven models offer a noninvasive solution, overcoming challenges of inter-tumor heterogeneity and providing more robust and unbiased PD-L1 scoring (15). Additionally, AI models, such as the TMBRB model, have been developed to predict the efficacy of ICIs in NSCLC by assessing TMB (16). Beyond radiomics, AI applications extend to pathological images, enabling the prediction of PD-L1 and TMB expression levels (17). Overall, AI-based imaging and predictive models are revolutionizing the personalized treatment of NSCLC, particularly in predicting the efficacy of immunotherapy, by offering more accurate and individualized predictions that inform clinical decision-making.

The tumor immune microenvironment (TIME), an intricate and dynamic ecosystem, is a critical determinant of both the cancer progression and response to immunotherapy (18). The TIME can either promote or inhibit anti-tumor immune responses, depending on the balance between immune-stimulatory and immune-suppressive elements within the microenvironment (19, 20). Recently, several AI-based approaches have been developed to analyze the TIME and quantify the immune infiltrate composition within tumors (21, 22). These approaches showed the potential to allow for a more full and unbiased evaluation of the TIME in comparison with standard approaches.

One of our previous studies has shown that Estimation of STromal and Immune cells in Malignant Tumors using Expression data (ESTIMATE) algorithm, which takes advantage of the unique properties of the transcriptional profiles of cancer samples to infer tumor cellularity and the infiltrating stromal and immune cells (23), could help to identify NSCLC patients who respond to chemoimmunotherapy. However, the application of gene signatures as biomarkers in clinical practice is challenging because their assessment is costly and inconvenient. Recent research has indicated the promise of pathomics to predict gene-based signatures (24, 25). However, the use of pathomics in identifying subsets of patients with NSCLC who are likely to benefit from chemo-immunotherapy has been less explored. Therefore, we investigated whether a pathomics-based ESTIMATE immunoscore could serve as a biomarker for chemoimmunotherapy in patients with advanced non-squamous NSCLC. We aimed to determine whether the AI-based immunoscore could predict response to treatment and clinical outcomes, such as progression-free survival (PFS) and overall survival (OS).

## Methods

### Study design and participants

**Figure 1:**
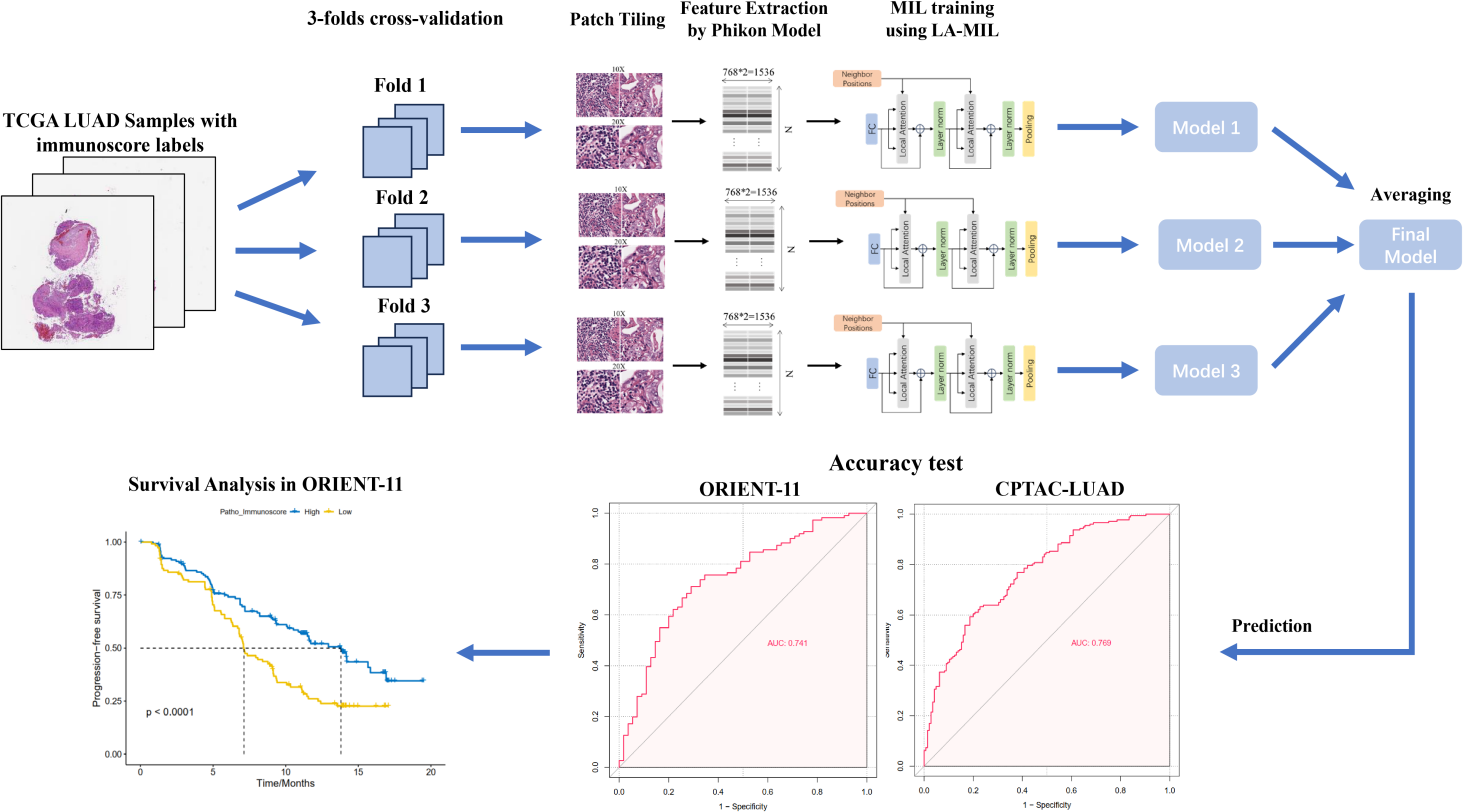
Workflow of the study. We developed the patho-immunoscore model with use of whole-slide scanned histology images and ESTIMATE-Immunoscore as the labels (determined by RNA sequencing) from TCGA-LUAD public data. The model was validated in two external series of surgical samples and lung biopsies, respectively. The predictive value of patho-immunoscore for chemoimmunotherapy was subsequently tested in the ORIENT-11 cohort. TCGA-LUAD, The Cancer Genome Atlas Lung Adenocarcinoma data collection; MIL, multiple instance learning.

The workflow of the study is displayed in **Figure 1**. Whole slide images (WSIs) from the The Cancer Genome Atlas Lung Adenocarcinoma (TCGA-LUAD) collection were used as training data, including both formalin-fixed paraffin-embedded (FFPE) and frozen section (FS) slides. FS slides were converted to FFPE style using the AI-FFPE method. We obtained a total of 1,333 WSIs from TCGA-LUAD and divided them into three folds for training and cross-validation. This dataset comprises adult patients with primary lung adenocarcinoma in the USA. The inclusion criteria were listed as followed (1): availability of digital Hematoxylin and Eosin (H&E)-stained histological slides from formalin-fixed paraffin-embedded (FFPE) samples (2); availability of gene expression profiling results based on RNA sequencing. Among the 585 patients in the entire series, 70 cases (11.97%) were excluded due to unavailability of whole-slide images from FFPE material or RNA sequencing data, resulting in the inclusion of 515 patients. External testing used cohorts from CPTAC-LUAD and ORIENT-11 study. The ORIENT-11 cohort was reanalyzed for survival analysis to validate therapy prediction efficacy. In the ORIENT-11 cohort, the immunochemotherapy group received sintilimab, an anti–programmed death-1 antibody, in combination with pemetrexed and platinum (cisplatin or carboplatin), while the control group received a placebo with pemetrexed and platinum (cisplatin or carboplatin). Detailed methods were described in **Supplementary Materials**.

### Image preprocessing

We dichotomized the ESTIMATE immune scores into high and low based on the median value of the TCGA-LUAD cohort (https://bioinformatics.mdanderson.org/estimate/) and applied it to both CPTAC-LUAD cohort and ORIENT-11 cohort (26). A weakly supervised multiple instance learning (MIL) approach was used to classify WSIs into high or low immune scores. Tissue regions were identified using thresholding, and image tiles were extracted at 20X and 10X magnifications. Features were encoded using the Phikon model and combined into a 1536-dimensional vector for each tile (27). We then used the Local self-attention graph-based transformer MIL method (LA-MIL) method for training, focusing on local attention among neighboring tiles (28). The model was trained using the AdamW optimizer, a learning rate of 2e-05, for 20 epochs. For the two external validation sets, the results from the three-fold cross-validation were aggregated using mean pooling to reduce the likelihood of misleading attention weight outcomes.

### Assessment of ESTIMATE score in the validation cohort

ESTIMATE leverages the distinctive characteristics of cancer sample transcriptional profiles to deduce the composition of tumor cellularity along with the presence of stromal and immune infiltrates (26). To validate the accuracy of our patho-immunoscore model, we calculated the ESTIMATE score in the CPTAC-LUAD cohort and the ORIENT-11 biomarker cohort. The RNA-seq FPKM data were retrieved from Linkedomics data portal (https://www.linkedomics.org/data_download/CPTAC-LUAD/). For bulk RNA sequencing of the ORIENT-11 study, the detailed protocols were displayed in **Supplementary methods**. RNA was extracted from tumor samples that were procured via core biopsy. Libraries were constructed using NEBNext Ultra II Directional RNA Library Prep Kit for Illumina. The sequencing was performed on the NovaSeq 6000 platform (Illumina). Gene expression data from the CPTAC-LUAD and ORIENT-11 cohorts were normalized by computing the transcripts per million (TPM) values. Then, we used the Immuno-Oncology Biological Research (IOBR) R package to calculate the ESTIMATE score (29).

### Assessment of PD-L1 protein levels through immunohistochemical staining

For the ORIENT-11 cohort, immunohistochemical analysis for the PD-L1 protein was performed on the baseline tumor specimens, which had been fixed with formalin and embedded in paraffin. The 22C3 pharmDx assay kit from Agilent Technologies was utilized, and the test was carried out at a centralized laboratory in Shanghai, China. We used the Tumor Proportion Score (TPS) to measure the expression level of PD-L1 in the tumor. This score reflects the proportion of tumor cells that display partial to full membrane staining for the protein, regardless of the staining intensity, as a percentage of the total viable tumor cells.

### Relationship between the patho-immunoscore and antigen presentation pathway

Building on our previous findings from the ORIENT-11 trial, where RNA sequencing data was used to establish the MHC class II antigen presentation pathway as a predictive biomarker for cancer immunotherapy (30), we further explored its relationship with the patho-immunoscore to identify the potential mechanisms of the predictive power of PIS in NSCLC immunotherapy. Similarly, we employed RNA sequencing to analyze the expression levels of 15 MHC class II-related genes (HLA-DMA, HLA-DMB, HLA-DOA, HLA-DOB, HLA-DPA, HLA-DPB1, HLA-DQA1, HLA-DQA2, HLA-DQB1, HLA-DQB2, HLA-DRA, HLA-DRB1, HLA-DRB3, HLA-DRB4, HLA-DRB5), which have been shown to highly corelated with NSCLC immunotherapy outcomes (30). To create a consistent, comparable measure across different genes, each gene’s expression was first normalized using z-scores, after which their averaged expression levels were calculated to reflect the overall activity of the MHC class II pathway (30). This approach allowed us to assess the association between antigen presentation and the patho-immunoscore more comprehensively, without being influenced by the absolute differences in expression levels among the 15 genes.

### Outcomes and statistical analysis

To assess the accuracy of patho-immunoscore models, receiver operating characteristic (ROC) curves, the area under the ROC curve (AUROC) and confusion matrix were used. The Kaplan-Meier method estimated median survival and created survival curves. Comparisons of PFS and OS between groups were conducted using hazard ratios (HRs) and 95% confidence intervals (CIs) obtained from the Cox regression model, with differences between groups in PFS or OS determined by the log-rank test. A multivariate Cox proportional hazards model was employed to analyze the interaction between treatment and patho-immunoscore, adjusting for age, gender, PD-L1 expression, BMI and smoking history. All statistical analyses were performed with R version 4.4.1 (R Core Team, Vienna, Austria), and a two-sided *p*-value was calculated.

## Results

### Study design and patient characteristics of the development cohort

The study design was displayed in **Figure 1**. We initially trained our model utilizing the publicly available TCGA-LUAD dataset. A total of 515 patients were included, and their main clinical, and pathological characteristics are summarised in the table (**Supplementary Table 1**). In the TCGA-LUAD cohort, 277 (53.79%) patients were female and 238 (46.21%) were female. The median age of diagnosis for the 496 patients with available data was 66 years (IQR,59–73). Race distribution of the patients was as follows: White, 388 (86.41%) of 449 with available data; Asian, 8 (1.78%); Black or African American, 52 (11.58%); and American Indian or Alaska Native, 1 (0.23%).

### Prediction accuracy of the patho-immunoscore model

**Figure 2:**
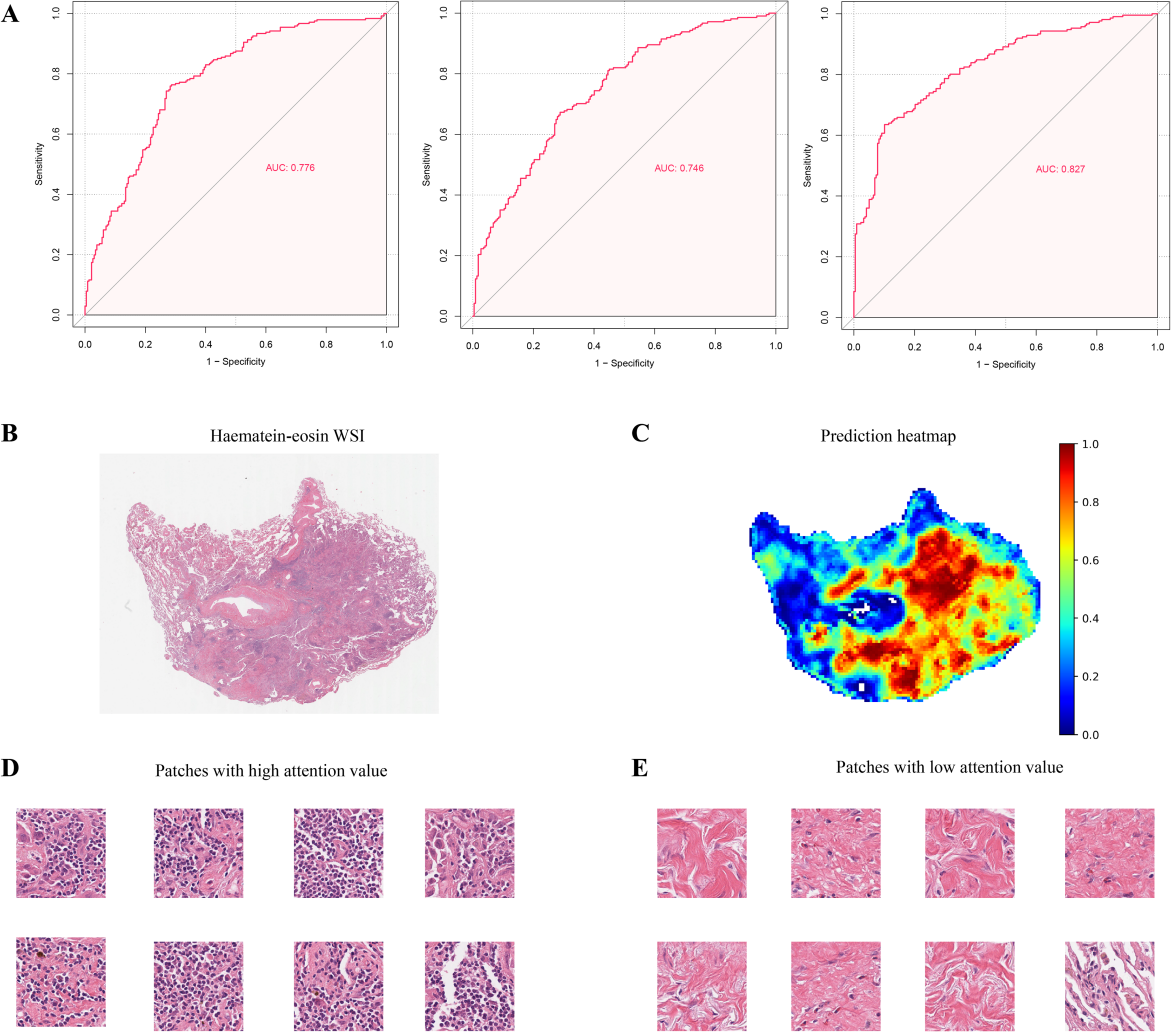
Prediction of Patho-immunoscore with deep learning. **(A)** The receiver operating characteristic (ROC) curves for the weakly supervised multiple instance learning model’s predictions of the patho-immunoscore in the TCGA-LUAD dataset. The model underwent three-fold cross-validation to assess its predictive performance, with each fold providing an independent evaluation. **(B, C)** Hematoxylin-eosin stained section **(B)** and the corresponding prediction heatmap **(C)** from a case within the development series test set are depicted, where high patho-immunoscore areas are marked in red and low patho-immunoscore areas in blue, with the model’s predictions scaled to a range of 0 to 1 **(D, E)**. Patches with high **(D)** and low **(E)** patho-immunoscore values: immune cell enrichment is observed in regions of high patho-immunoscore.

The model was trained using a three-fold cross-validation approach. The accuracy of the model was assessed by calculating the Area Under the Curve (AUC) for each fold. The results showed that the model achieved an AUC of 0.776 in Fold 1, 0.746 in Fold 2, and 0.827 in Fold 3 (**Figure 2A**), achieving a mean AUC of 0.783 in the TCGA-LUAD cohort. These findings demonstrated the model’s robust predictive performance and reliability. The model’s predictive efficacy was further validated in the CPTAC-LUAD cohort and the biomarker cohort of the ORIENT-11 study, a randomized, double-blind, phase 3 trial. Significantly, the external datasets exhibited variances from the development cohort concerning ethnic backgrounds, sampling methods, and staining procedures.

The first validation dataset included 106 patients treated by surgical resection in the CPTAC project (**Supplementary Table 2**). The samples were obtained from patients diagnosed with LUAD who underwent surgical resection without receiving any neoadjuvant therapy. The CPTAC-LUAD cohort comprised 68 (64.15%) male patients and 38 (35.75%) female patients. Different from the TCGA-LUAD cohort, the majority of the CPTAC-LUAD cohort were Asian or Han Ethnicity (59/103 with available data, 57.28%), with Caucasian or European Ethnicity accounting for 38.83% (40/103). Other ethnicities like Black, White, Hispanic account for 3.9% (4/103).

Given that biopsy is the major sample source for patients with advanced non-squamous NSCLC, we proceeded to validate the accuracy of our patho-immunoscore model in the ORIENT-11 cohort, an external dataset that only included biopsies. In the ORIENT-11 study, 397 participants diagnosed with stage IIIB-IV non-squamous NSCLC were enrolled and randomly assigned to the chemoimmunotherapy groups (n = 266) and chemotherapy groups (n = 131). A total of 387 participants have available H&E pathology slides that are compliant with quality control and were finally included in this study (**Supplementary Table 3**), among whom 166 patients have available RNAseq data, including 110 patients treated with chemoimmunotherapy and 56 patients treated with chemotherapy only. All the participants in the ORIENT-11 cohort were Asian, among whom 92 (23.77%) patients were female and 295 (76.23%) patients were male.

Our immunoscore analyzer demonstrated robust accuracy across the two validation cohorts, with an AUC of 0.741 in the ORIENT-11 cohort, and an AUC of 0.769 in the CPTAC-LUAD cohort, respectively (**Supplementary Figure 1**). The confusion matrices of these models were displayed in **Supplementary Figure 2**. These results underscore the potential of our immunoscore analyzer as a reliable tool for predictive modeling in non-squamous NSCLC. Of note, the pathological examination of image patches that were predicted to have high ESTIMATE immunoscore values (high PIS values) revealed a notable presence of immune cells (**Figures 2B–E**).

### Patho-immunoscore and clinical outcome

For the ORIENT-11 cohort, the baseline clinical characteristics, PFS and OS were well balanced between pathomics-evaluable population and intent-to-treat cohorts (**Supplementary Tables 3, 4** and **Supplementary Figure 3**). Besides, the baseline clinical characteristics were balanced between chemoimmunotherapy group and chemotherapy group. Among the 387 individuals in the ORIENT-11 cohort included in our study, the median follow-up for progression-free survival was 13.9 months, with an IQR of 13.7 to 14 months. For overall survival, the median follow-up time was 31.2 months, with an IQR ranging from 30.8 to 32 months. Of these, 214 patients (55.3%), had tumors that were rated as high PIS, while 173 patients (44.7%), were noted to have low PIS tumors.

**Figure 3:**
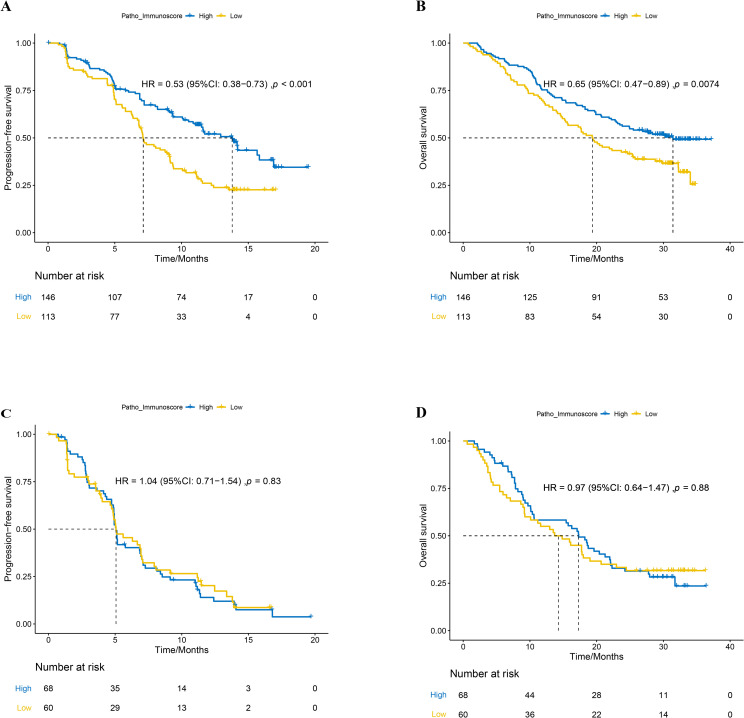
The relationship between patho-immunoscore and survival outcome in the ORIENT-11 cohort. **(A)** Progression-free survival for combination therapy-treated patients according to the status of patho-immunoscore. **(B)** Overall survival for combination therapy-treated patients according to the status of patho-immunoscore. **(C)** Progression-free survival for chemotherapy-treated patients according to the status of patho-immunoscore. **(D)** Overall survival for chemotherapy-treated patients according to the status of patho-immunoscore.

In a cohort of 259 patients treated with chemoimmunotherapy (combo group), those with a high patho-immunoscore (n=146) exhibited significantly longer progression-free survival (PFS) compared to those with a low patho-immunoscore (n=113), with median PFS times of 13.8 months versus 7.13 months, respectively (**Figure 3A**, hazard ratio [HR]: 0.53, 95% confidence interval [CI]: 0.38 – 0.73; *p* < 0.001). To determine if PIS serves as a universal indicator of patient outcomes in NSCLC or as a distinctive biomarker for predicting responses to chemoimmunotherapy, we examined the efficacy of first-line chemotherapy regimens in the chemotherapy group of ORIENT-11 study. Conversely, among patients treated with chemotherapy alone, there was no significant difference in PFS between the high patho-immunoscore and low patho-immunoscore groups, with median PFS times of 5.7 months (95% CI: 4.73 months – 7.00 months) versus 5.7 months (95% CI: 4.90 months – 6.93 months), respectively (**Figure 3C**, HR: 1.04, 95% CI: 0.71 – 1.54; *p* = 0.83).

Similarly, the overall survival (OS) was significantly longer in the combo group for patients with a high PIS compared to those with a low PIS, with median OS times of 31.43 months (95% CI: 23.47 months – not reached) versus 19.40 months (95% CI: 15.33 months – 25.47 months), respectively (**Figure 3B**, HR: 0.65, 95% CI: 0.47 – 0.89; *p* < 0.001). In the chemotherapy-only group, there was no significant difference in OS between the high and low PIS groups, with median OS times of 17.30 months (95% CI: 9.23 months – 20.67 months) versus 14.27 months (95% CI: 10.6 months – 22.07 months), respectively (**Figure 3D**, HR: 0.97, 95% CI: 0.64 – 1.47; *p* = 0.88).

**Figure 4:**
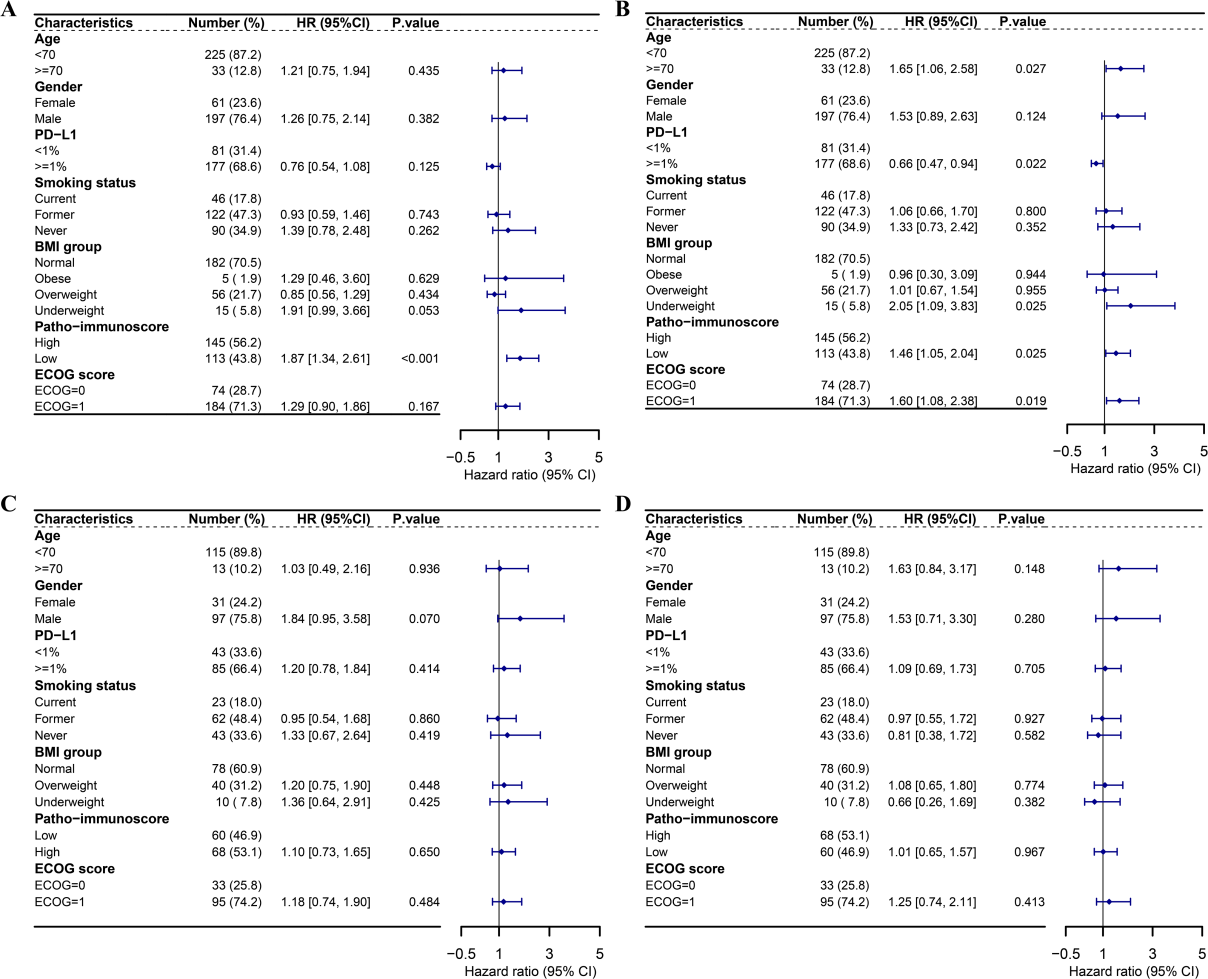
Multivariate Cox regression analyses of survival outcomes in the ORIENT-11 cohort. **(A)** Forest plot showing multivariate Cox regression analysis of the effect of patho-immunoscore and other clinical characteristics on PFS of patients who received combination therapy in the ORIENT-11 cohort. **(B)** Forest plot showing multivariate Cox regression analysis of the effect of patho-immunoscore and other clinical characteristics on OS of patients who received combination therapy in the ORIENT-11 cohort. **(C)** Forest plot showing multivariate Cox regression analysis of the effect of patho-immunoscore and other clinical characteristics on PFS of patients who received chemotherapy in the ORIENT-11 cohort. **(D)** Forest plot showing multivariate Cox regression analysis of the effect of patho-immunoscore and other clinical characteristics on OS of patients who received chemotherapy in the ORIENT-11 cohort. BMI, body mass index; CI, confidence interval; ECOG, Eastern Cooperative Oncology Group; HR, hazard ratio; PD-L1, programmed death-ligand 1; PFS, progression-free survival; OS, overall survival.

Furthermore, multivariate regression analyses were conducted to evaluate the PIS alongside other clinical characteristics. Considering that only one case of baseline BMI data was not available in the combination group, we adopted the listwise deletion strategy and included 258 cases in the multivariate regression analyses for the combination group (31). In the combination group, a significant correlation was observed between the PIS and improved PFS (HR = 0.54, 95% CI: 0.38–0.75, *p* < 0.001) as well as OS (HR = 0.68, 95% CI: 0.49–0.95, *p* = 0.025) (**Figures 4A, B**). Considering the PIS and additional clinical factors, no significant link was found between PD-L1 and PFS (HR = 0.76, 95% CI: 0.54–1.08, *p* = 0.125). Nevertheless, patients with positive PD-L1 expression (TPS ≥ 1%) appeared to gain more overall survival benefits from chemoimmunotherapy compared to those with negative PD-L1 expression (HR = 0.66, 95% CI: 0.47–0.94, *p* = 0.022). Underweight patients, in contrast to those of normal weight, exhibited reduced overall survival benefits from chemoimmunotherapy (HR = 2.05, 95% CI: 1.09–3.83, *p* = 0.025). Additionally, age and ECOG status were found to be influential in predicting the overall survival of patients undergoing chemoimmunotherapy (**Figure 4B**). Conversely, in the chemotherapy group, no significant association was identified between the PIS and survival outcomes, nor with other clinical factors mentioned earlier (**Figures 4C, D**).

### Combination of PD-L1 and patho-immunoscore

**Figure 5:**
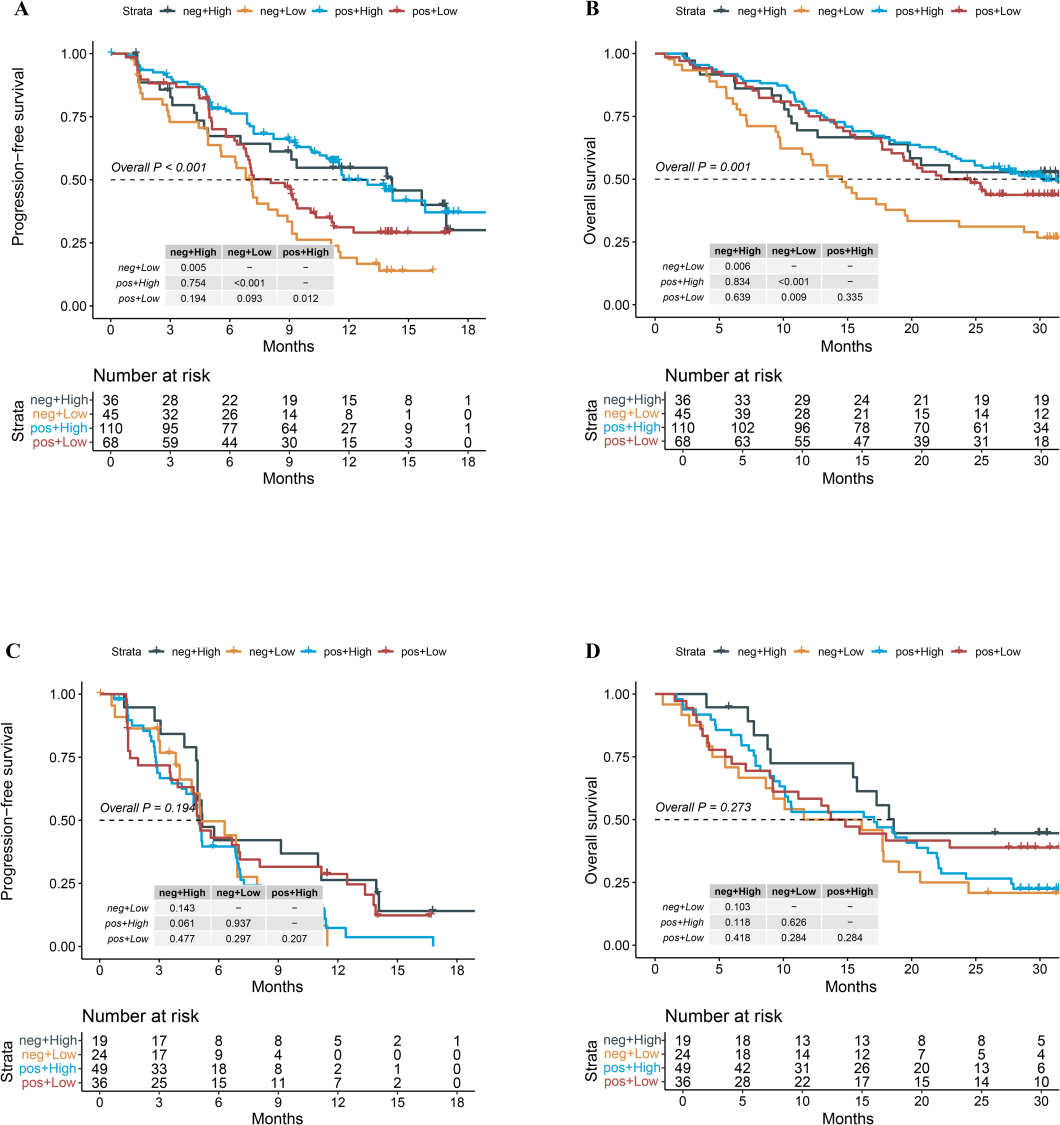
Survival outcomes of the combination group and chemotherapy group stratified by the status of PD-L1 expression and patho-immunoscore level. **(A)** Kaplan-Meier analysis of PFS grouped by PD-L1 status and patho-immunoscore level in the combo group of the ORIENT-11 cohort. **(B)** Kaplan-Meier analysis of OS grouped by PD-L1 status and patho-immunoscore level in the combo group of the ORIENT-11 cohort. **(C)** Kaplan-Meier analysis of PFS grouped by PD-L1 status and patho-immunoscore level in the chemotherapy group of the ORIENT-11 cohort. **(D)** Kaplan-Meier analysis of OS grouped by PD-L1 status and patho-immunoscore level in the chemotherapy group of the ORIENT-11 cohort. OS, overall survival; PD-L1, programmed death-ligand 1; PFS, progression-free survival; Combo, combination therapy of immunotherapy and chemotherapy.

Further, we explored whether the combination of PD-L1 and PIS could help us find patients who would gain more survival benefit from chemoimmunotherapy. As shown in **Figure 5A**, no matter whether PD-L1 was positive or negative, patients with high PIS tended to have significantly longer PFS than those with low PIS in the combo settings (**Figure 5A**, *p* < 0.05). In those who have low PIS, the PFS of patients with positive PD-L1 expression was numerically longer than patients with negative PD-L1 expression in the combo settings (**Figure 5A**, *p* = 0.093). Interestingly, we found that patients with positive PD-L1 expression tended to gain more overall survival benefit from chemoimmunotherapy even though the PIS were low (**Figure 5B**, *p* = 0.009). In contrast, neither PD-L1 nor PIS was associated with the survival outcomes of patients who received chemotherapy only (**Figures 5C, D**).

### Patho-immunoscore correlated with MHC-II

In our prior investigation within the ORIENT-11 study, we observed a link between the activity of the MHC class II antigen presentation mechanism and the benefits observed in terms of progression-free survival (PFS) and overall survival (OS) for the treatment combination (30). In this study, we further examined the association between MHC class II antigen presentation and the PIS, using the CPTAC cohort as well as the ORIENT-11 cohort, as depicted in **Supplementary Figure 4**. Notably, patients with high PIS exhibited higher MHC class II antigen presentation activity (*P* < 0.05), suggesting that high PIS is associated with activated immunity and “hotter” tumor immune microenvironment.

## Discussion

Our previous study has shown that patients with both high PD-L1 expression and high ESTIMATE immune score tended to gain more survival benefit from chemotherapy plus immunotherapy (23). However, RNA sequencing is not convenient and cost-effective in routine clinical practice. Therefore, in this study, we developed a pathomics-based immunoscore model using data from TCGA. By leveraging pathomics from whole-slide images, our immunoscore analyzer demonstrated robust predictive accuracy across three independent cohorts (TCGA-LUAD, ORIENT-11, and CPTAC-LUAD). Further, our multicentre retrospective study highlights the significant potential of an AI-powered immunoscore in predicting the efficacy of chemoimmunotherapy for patients with advanced non-squamous NSCLC. As far as we know, this study is one of the first to assess a genomic signature biomarker with pathomics data in non-squamous NSCLC, and the first to validate the relative predictive value for chemoimmunotherapy in a phase III randomized controlled trial cohort. In addition, using patients receiving chemotherapy as control, our study suggested that patho-immunoscore could serve as a predictive biomarker for immunotherapy instead of a prognostic biomarker for advanced NSCLC patients.

Over the past few years, multiplex tissue imaging methods have provided in-depth profiling of the tumor microenvironment (TME) for patients (32–36). However, their extensive cost has restricted their broad application in clinical practice (37). Conversely, HE-stained pathology slides offer a cost-effective and accessible alternative, commonly found in pathology laboratories. These slides harbor extensive data related to the TME, which can be deciphered through the application of artificial intelligence (38). By utilizing the AI in HE-stained slides to generate the PIS status, our study offers a reliable, repeatable pre-treatment predictor of ICIs treatment, thereby facilitating the clinical application of personalized management for patients diagnosed with advanced non-squamous NSCLC. In previous pathomics analyses, the main emphasis has been on the density and space distribution of tumor-infiltrating lymphocytes (TILs) (39–42). For example, the Hover-Net system was a neural network system that was capable of segmenting and classifying nuclei across various cancers, facilitating detailed single-cell analysis of tumor cells, stroma cells, and lymphocytes from H&E slides (43). Pioneering large-scale TME characterization from H&E slides was initiated by Abousamra and colleagues, who cataloged the prevalence and spatial arrangement of tumor-infiltrating lymphocytes (TILs) in 23 distinct cancer types (44). Further, Sehhoon Park and colleagues developed an AI–powered spatial analyzer for TILs in NSCLC (41). This tool can identify three immune phenotypes (IPs): inflamed, immune-excluded, and immune-desert. These IPs are correlated with the response to ICIs in patients diagnosed with NSCLC, potentially optimizing treatment selection in clinical practice for advanced NSCLC. Similarly, another study conducted by Rakaee et al. also revealed that machine-learning-based TILs assessment could be a valuable tool for predicting the response to ICI therapy in NSCLC patients, particularly in those with PD-L1 negative status (42). Although these methods are invaluable, they are impeded by the requirement for meticulous annotations provided by expert pathologists, a procedure that is inherently laborious and resource-intensive. To counteract these constraints, various research teams have suggested employing weakly-supervised deep learning models. These models are capable of accomplishing diverse computational pathology tasks, including tumor subtyping and outcome prediction, without the need for precise region or pixel-level annotations (25, 45–47). Similar to these studies, our study adopted a weakly supervised multiple instance learning (MIL) approach to train the patho-immunoscore model (48). Despite the inherent risk of overfitting in deep learning models, our research excels in its broad external validation. Our model has been put to the test in various settings, including different centers, diverse staining protocols, and ethnicities. In addition, the fixed threshold applied to the model showed possible clinical implementation potential for individual patient categorization since the results are encouraging.

The current clinical practice often relies on PD-L1 expression, TMB and Microsatellite instability (MSI) as biomarkers to predict immunotherapy efficacy (49–53). However, the prediction efficacies of these biomarkers are still limited. Our patho-immunoscore model, which integrates comprehensive histological and immune microenvironment data, offers a more nuanced and potentially more accurate prediction of chemoimmunotherapy efficacy. This advantage is particularly evident in our study, where the patho-immunoscore provided significant stratification of patient outcomes, independent of PD-L1 status.

Despite the promising results, our study has several limitations. As a retrospective analysis, the findings require prospective validation to confirm their clinical utility. The variability in sample processing and staining across different cohorts may introduce biases, although our model’s robust performance across diverse datasets mitigates this concern to some extent. Additionally, while our study focused on non-squamous NSCLC, further research is needed to determine the patho-immunoscore’s applicability to squamous cell carcinoma and other histological subtypes. Finally, efforts should be made to improve the interpretability of the ensemble model. We have conducted TME analysis pertaining to model predictions and found that patho-immunoscore was correlated with MHC-II pathway activation. However, we have not explored the correlation between pathomics features and molecular mechanisms. Future investigations aimed at comprehending the underlying mechanisms of these pathomics features and their performance will aid in establishing causal patho-immunogenomic relationships, thereby unraveling the biological intricacies that drive ensemble prediction.

In conclusion, our study provides compelling evidence that an AI-powered immunoscore based on pathomics can serve as a robust biomarker for predicting the efficacy of chemoimmunotherapy in patients with advanced non-squamous NSCLC. Despite the limitations posed by the lack of further clinical dataset validation, the model’s performance within the phase-III randomized controlled trial cohort for advanced non-squamous NSCLC provides compelling evidence of its potential to improve patient selection for immunotherapy. This approach not only enhances our ability to personalize cancer treatment but also opens new avenues for the application of AI in oncological research and clinical practice. By improving the precision of treatment selection, the patho-immunoscore holds the potential to significantly impact patient outcomes and advance the field of cancer immunotherapy.

## Data Availability

The data analyzed in this study is subject to the following licenses/restrictions: The data of TCGA-LUAD cohort and CPTAC-LUAD cohort were publicly available as described in “Methods” and “Supplementary” sections. Data from ORIENT-11 cohorts cannot be made available due to privacy and ethical or legal issues. Requests to access these datasets should be directed to zhangli6@mail.sysu.edu.cn.
