# Supplementary material for: Association of artificial intelligence-based immunoscore with the efficacy of chemoimmunotherapy in patients with advanced non-squamous non-small cell lung cancer: a multicentre retrospective study
Source: Front Immunol. 2024 Nov 6;15:1485703. doi: 10.3389/fimmu.2024.1485703 (PMC11576461; doi:10.3389/fimmu.2024.1485703)
Supplement: Supplementary file 1 [file DataSheet1.pdf]

## Supplementary Methods

### Image Processing

Whole slide images (WSIs) from The Cancer Genome Atlas Lung Adenocarcinoma (TCGA-LUAD) collection were used as training data. To enhance the dataset, we included WSIs from both formalin-fixed paraffin-embedded (FFPE) diagnostic slides and frozen sectioned (FS) tissue slides. Due to the typically lower quality of FS slides for downstream analysis, we first converted FS slides to FFPE style using the AI-FFPE method, a generative deep learning model designed for style transformation between FS and FFPE images (1). We utilized the pretrained weights for lung images provided in the AI-FFPE study. In total, we obtained 1,333 WSIs from TCGA-LUAD and randomly divided them into three folds for training and cross-validation. For external testing, we used two cohorts: one from the Clinical Proteomic Tumor Analysis Consortium Lung Adenocarcinoma (CPTAC-LUAD) collection and another from a phase-III clinical trial (ORIENT-11). ESTIMATE immune scores were obtained from https://bioinformatics.mdanderson.org/estimate/ and dichotomized into high and low based on the median value of the entire TCGA-LUAD cohort. The same cutoff value was applied to dichotomize the ESTIMATE immune scores for CPTAC-LUAD and ORIENT-11.

To train a classification model to assign a WSI to either a high or low immune score, we employed a weakly supervised multiple instance learning (MIL) approach due to the large size of WSIs, which are typically too extensive for standard image classification models. First, we identified tissue regions in the WSIs using a simple thresholding method and generated a set of image tiles from these regions. The tiles were extracted at two magnifications (20X: 0.5 µm/pixel, 10X: 1 µm/pixel) with dimensions of 224 x 224 pixels to incorporate information from multiple scales. We used the Phikon model (2), a recently released foundation model pretrained on over 40 million pancancer digital pathology images, as the feature extractor to encode each image tile into a 768-dimensional feature vector. The features from both magnifications were then combined to form a 1536-dimensional feature vector for each tile. Next, we applied a local self-attention graph-based transformer MIL method (LA-MIL) for weakly supervised training based on the extracted features (3). Unlike classical MIL methods, which often neglect spatial contextual information between image tiles, transformer-based MIL methods can naturally model the interdependencies among all instances through their intrinsic self-attention mechanism.

However, global self-attention might not be necessary for histopathology images, particularly for biopsy samples where tissues might be fragmented, and spatial relations between tiles may not be real. Therefore, LA-MIL focuses only on local attention among neighboring image tiles, which is more suitable for our application. We followed the original implementation for most training parameters(3), including the AdamW optimizer, a learning rate of 2e-05 for 20 epochs, batch size of 1, and weighted binary cross-entropy loss. The number of neighboring tiles was set to 16 and 64 for local attention restriction in the first and second attention modules, respectively.

Training was performed for each fold, and the F1 score was calculated on the validation set for each epoch to identify the best weight. The three-fold training and cross-validation produced three different models, each yielding a probability output. During the training of the MIL model, the Phikon model served as a pre-trained feature extractor, embedding each patch into a feature vector.

To be noted, Phikon was recognized as one of the leading foundation models, considering training data size and model complexity. We also compared Phikon with other models such as Retccl (4), Pathduet (5), and BROW (6) in a three-fold cross-validation on the TCGA dataset. Our preliminary findings indicated no significant differences among the models, with Phikon performing slightly better overall, the AUC of which is shown in Supplementary Table 5. Therefore, we finally chose the Phikon model in this study. We averaged the probability values from the three models to obtain the final prediction results, which were then tested on the CPTAC-LUAD and ORIENT-11 cohorts. Finally, survival analysis was performed on the ORIENT-11 cohort to validate the efficacy of therapy prediction.

**RNA sequencing in ORIENT-11 cohort**

In the ORIENT-11 trial, only core biopsies were used to gather samples of tumors. The baseline FFPE tumor samples were extracted of their RNA using the RNeasy kit for such specimens, and then ribosomal RNA was removed by the NEBNext rRNA Depletion kit (New England Biolabs, NEB). Libraries were prepared using the NEBNext Ultra II Directional RNA Library Prep Kit for Illumina based on manufacturer's protocol. The method started with divalent cation induced fragmentation, continued through strand-specific construction of RNA-seq libraries. NovaSeq 6000 (Illumina) was utilized for sequencing. Read quality was verified with FastQC (4), and raw reads were trimmed using Trimmomatic before aligning to the human GRCh38 reference genome followed by alignment against STAR (Spliced Transcripts Alignment to a Reference) (5,6). We selected only samples with a unique read mapping rate > 30% and read duplication < 90% for downstream analysis. The number of reads aligned to specific genes was quantified using the HTSeq-count utility (7). Raw read counts were normalized as transcripts per million (TPM) to consider gene length and raw read count.

**Reference**


1. Ozyoruk KB, Can S, Darbaz B, Başak K, Demir D, Gokceler GI, Serin G, Hacisalihoglu UP, Kurtuluş E, Lu MY, et al. A deep-learning model for transforming the style of tissue images from cryosectioned to formalin-fixed and paraffin-embedded. Nat Biomed Eng (2022) 6:1407–1419.
2. Filiot A, Ghermi R, Olivier A, Jacob P, Fidon L, Mac Kain A, Saillard C, Schiratti J-B. Scaling self-Supervised Learning for histopathology with Masked Image Modeling. bioRxiv (2023) doi: 10.1101/2023.07.21.23292757
3. Reisenbüchler D, Wagner SJ, Boxberg M, Peng T. Local Attention Graph-Based Transformer for Multi-target Genetic Alteration Prediction. Medical Image Computing and Computer Assisted Intervention – MICCAI 2022. Springer Nature Switzerland (2022). p. 377–386
4. Wang X, Du Y, Yang S, Zhang J, Wang M, Zhang J, Yang W, Huang J, Han X. RetCCL: Clustering-guided contrastive learning for whole-slide image retrieval. Med Image Anal (2023) 83:102645.
5. Hua S, Yan F, Shen T, Ma L, Zhang X. PathoDuet: Foundation models for pathological slide analysis of H&E and IHC stains. Med Image Anal (2024) 97:103289.
6. Wu Y, Li S, Du Z, Zhu W. BROW: Better featuRes fOr Whole slide image based on self-distillation. arXiv [csCV] (2023) doi: 10.48550/ARXIV.2309.08259
7. de Sena Brandine G, Smith AD. Falco: high-speed FastQC emulation for quality control of sequencing data. F1000Res (2019) 8:1874.

8. Bolger AM, Lohse M, Usadel B. Trimmomatic: a flexible trimmer for Illumina sequence data. Bioinformatics (2014) 30:2114–2120.
9. Dobin A, Davis CA, Schlesinger F, Drenkow J, Zaleski C, Jha S, Batut P, Chaisson M, Gingeras TR. STAR: ultrafast universal RNA-seq aligner. Bioinformatics (2013) 29:15–21.
10. Anders S, Pyl PT, Huber W. HTSeq--a Python framework to work with high-throughput sequencing data. Bioinformatics (2015) 31:166–169.

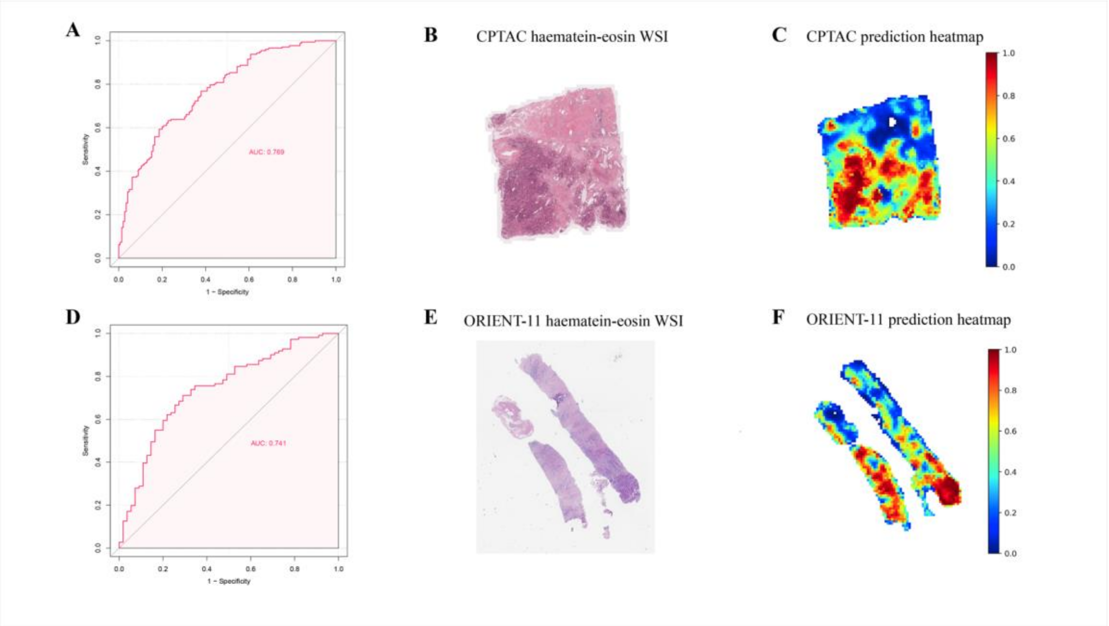


**Supplementary Figure 1. External validation of the patho-immunoscore model.** The receiver operating characteristic (ROC) curves for the weakly supervised multiple instance learning model's predictions of the patho-immunoscore in the CPTAC-LUAD cohort (A) and ORIENT-11 cohort (D). Haematein-eosin stained section and the corresponding prediction heatmap for a sample in the CPTAC-LUAD cohort (B-C) and a sample in the ORIENT-11 cohort (E-F). The heatmaps utilize red to denote areas of high patho-immunoscore and blue for areas of low patho-immunoscore values.

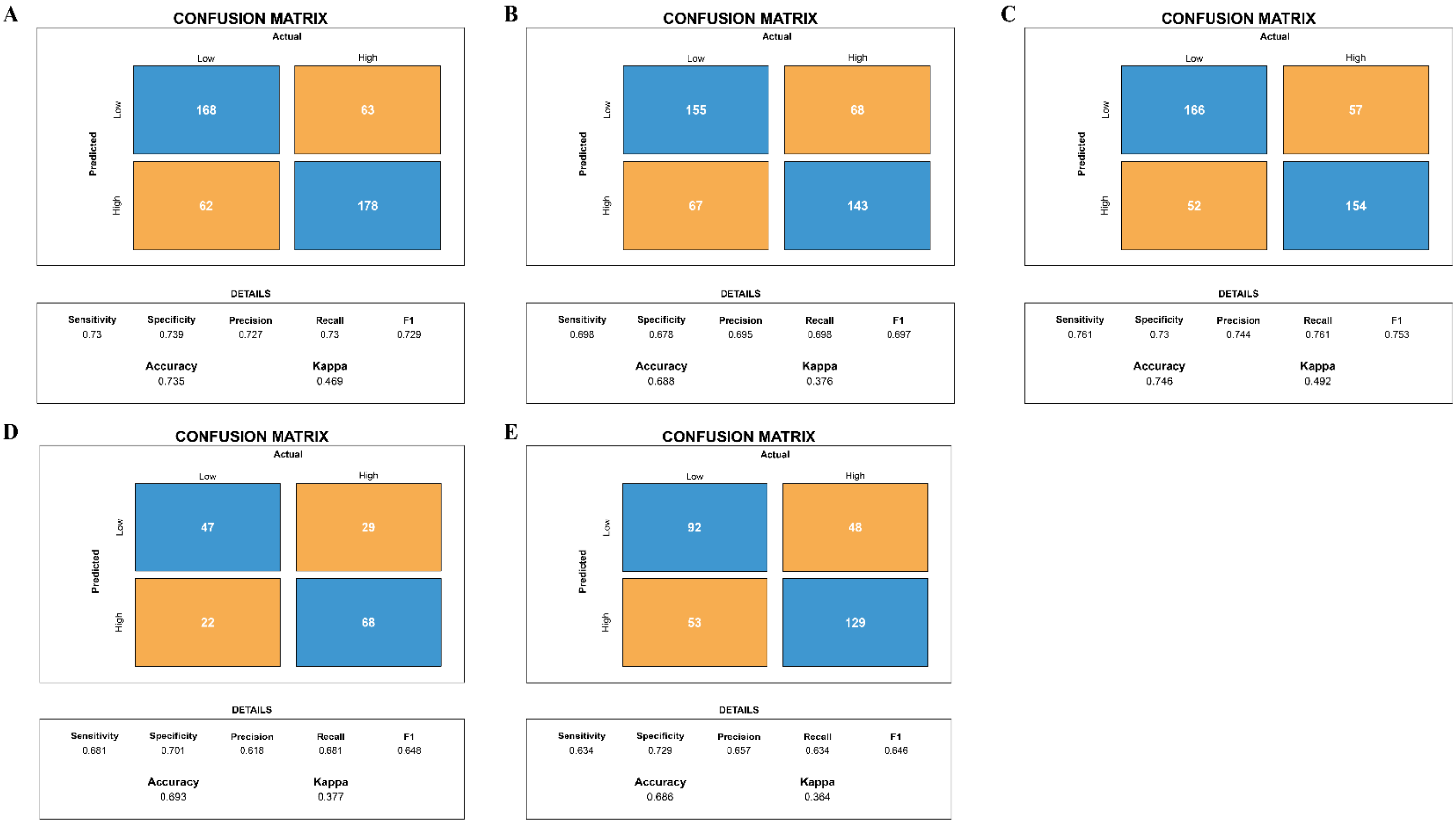


**Supplemental Figure 2**. Confusion matrix of the patho-immunoscore model across different datasets. **A-C.** Confusion matrices showing the classification performance of the patho-immunoscore model on the three folds of the TCGA-LUAD dataset. **D.** Confusion matrix summarizing the model's performance on the ORIENT-11 dataset. **E.** Confusion matrix depicting the accuracy of the patho-immunoscore model when applied to the CPTAC-LUAD dataset.

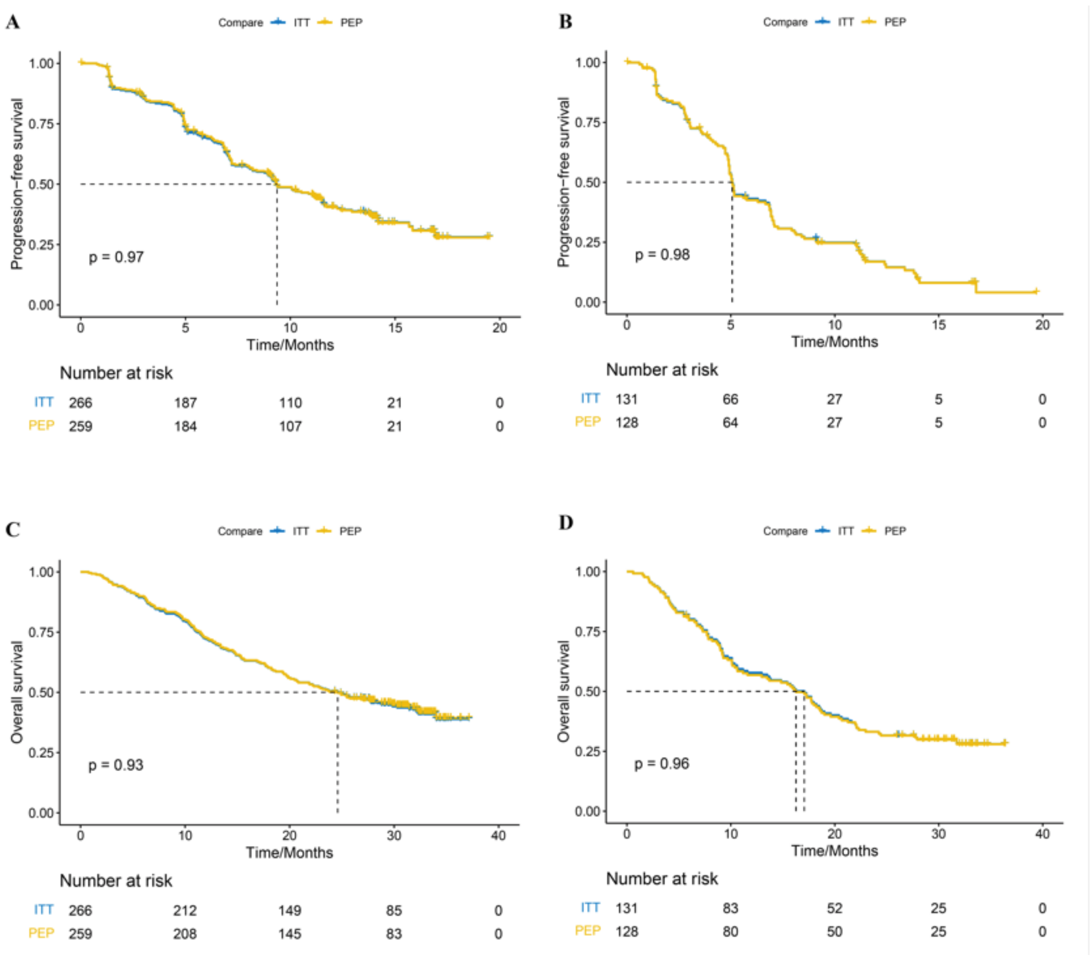


**Supplemental Figure 3**. Progression-free and OS Survival Analyses Between ITT and PEP in Combination Group and Chemotherapy Group. A, PFS was well balanced between the PEP and ITT cohorts in patients who received combination therapy. B, PFS was well balanced between the PEP and ITT cohorts in patients who received chemotherapy only. C, OS was well balanced between the PEP and ITT cohorts in patients who received combination therapy. D, FS was well balanced between the PEP and ITT cohorts in patients who received chemotherapy only. PFS, progression-free survival; OS, overall survival; ITT, intent-to-treat; PEP, pathomics evaluable population.

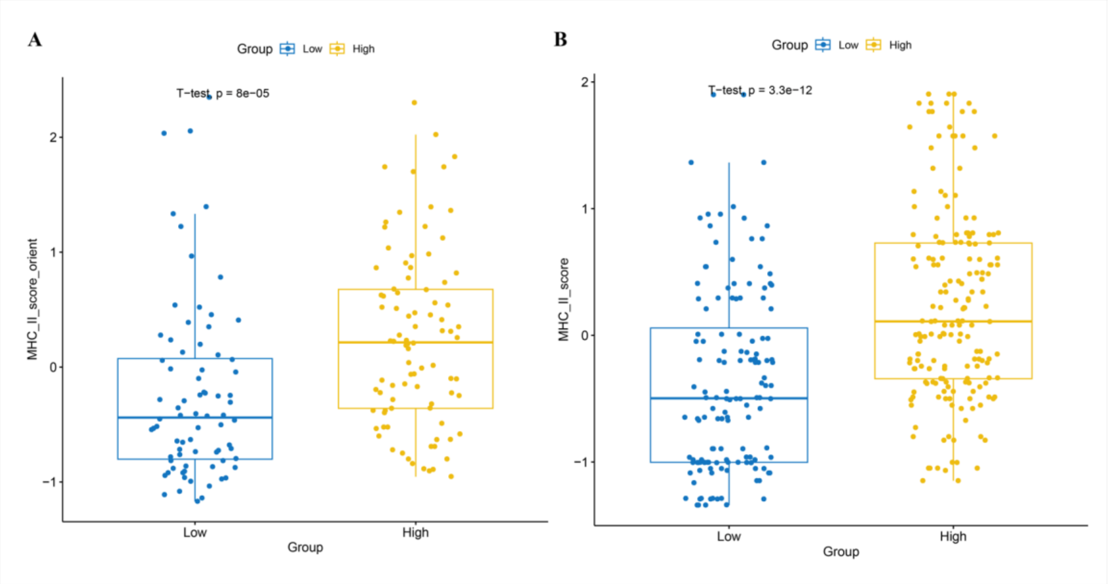


**Supplemental Figure 4**. Relationship between patho-immunoscore and MHC Class II Antigen Presentation Pathway in the ORIENT-11 cohort (A) and CPTAC-LUAD cohort (B). MHC, major histocompatibility complex.

**Supplemental Table 1. Patient Characteristics of TCGA-LUAD cohort**

| Variable | Female | Male |
| --- | --- | --- |
| N | 277 | 238 |
| Smoking history | | |
| Ever | 215 (77.6) | 211 (88.7) |
| Never | 55 (19.9) | 20 (8.4) |
| Unknown | 7 (2.5) | 7 (2.9) |
| Stage | | |
| Stage I | 161 (58.1) | 114 (47.9) |
| Stage II | 55 (19.9) | 67 (28.2) |
| Stage III | 46 (16.6) | 38 (16.0) |
| Stage IV | 12 (4.3) | 14 (5.9) |
| Unknown | 3 (1.1) | 5 (2.1) |
| Race | | |
| AMERICAN INDIAN OR ALASKA NATIVE | 1 (0.4) | 0 (0.0) |
| ASIAN | 4 (1.4) | 4 (1.7) |
| BLACK OR AFRICAN AMERICAN | 29 (10.5) | 23 (9.7) |
| WHITE | 215 (77.6) | 173 (72.7) |
| Unknown | 28 (10.1) | 38 (16.0) |
| Diagnosis Age ≥70 | 105 (39.3) | 86 (37.6) |

**Supplemental Table 2. Patient Characteristics of CPTAC-LUAD cohort**

| Variable | Female | Male |
|---|---|---|
| N | 38 | 68 |
| Stage | | |
| Stage I | 15 (39.5) | 29 (42.6) |
| Stage II | 6 (15.8) | 11 (16.2) |
| Stage III | 2 (5.3) | 9 (13.2) |
| Unknown | 15 (39.5) | 19 (27.9) |
| Ethnicity | | |
| Asian | 12 (31.6) | 29 (42.6) |
| Black | 1 (2.6) | 0 (0.0) |
| Caucasian | 13 (34.2) | 22 (32.4) |
| European | 2 (5.3) | 3 (4.4) |
| Han | 7 (18.4) | 11 (16.2) |
| Hispanic | 1 (2.6) | 1 (1.5) |
| White | 0 (0.0) | 1 (1.5) |
| Unknown | 2 (5.3) | 1 (1.5) |
| BMI | | |
| Normal | 21 (55.3) | 28 (41.2) |
| Obese | 7 (18.4) | 5 (7.4) |
| Overweight | 7 (18.4) | 21 (30.9) |
| Underweight | 3 (7.9) | 14 (20.6) |
| Region of Origin (Western) | 20 (52.6) | 28 (41.2) |
| Smoking Status | | |
| Non-smoker | 22 (57.9) | 21 (30.9) |
| Smoker | 14 (36.8) | 41 (60.3) |
| Unknown | 2 (5.3) | 6 (8.8) |

**Supplemental Table 3. Patient Characteristics of ORIENT-11 cohort**

| Variable | Combo[a] ITT | Combo[a] PEP | Chemo ITT | Chemo PEP |
|---|---|---|---|---|
| N | 266 | 259 | 131 | 128 |
| Age ≥70 (%) | 34 (12.8) | 33 (12.7) | 13 (9.9) | 13 (10.2) |
| BMI (%) | | | | |
| Normal | 188 (70.7) | 182 (70.3) | 79 (60.3) | 78 (60.9) |
| Obese | 5 (1.9) | 5 (1.9) | 0 (0.0) | 0 (0.0) |
| Overweight | 57 (21.4) | 56 (21.6) | 42 (32.1) | 40 (31.2) |
| Underweight | 15 (5.6) | 15 (5.8) | 10 (7.6) | 10 (7.8) |
| Unknown | 1 (0.4) | 1 (0.4) | 0 (0.0) | 0 (0.0) |
| PD-L1 ≥1% (%) | 182 (68.4) | 178 (68.7) | 87 (66.4) | 85 (66.4) |
| ECOG score ECOG=1 (%) | 190 (71.4) | 185 (71.4) | 97 (74.0) | 95 (74.2) |
| Smoking status (%) | | | | |
| Current | 49 (18.4) | 46 (17.8) | 23 (17.6) | 23 (18.0) |
| Former | 122 (45.9) | 122 (47.1) | 64 (48.9) | 62 (48.4) |
| Never | 95 (35.7) | 91 (35.1) | 44 (33.6) | 43 (33.6) |

Abbreviations: BMI, body mass index; ECOG, Eastern Cooperative Oncology Group; PD-L1, programmed death-ligand 1; ITT, intent-to-treat; PEP, pathomics evaluable population

[a] Patients treated with sintilimab combined with platinum-based chemotherapy.

**Supplemental Table 4. Baseline Characteristics of Pathomics Evaluable Population Stratified by Treatment Received**

| Variable | Combo[a] | Chemo | *P*-value |
|---|---|---|---|
| N | 259 | 128 | |
| Age >=70 (%) | 33 (12.7) | 13 (10.2) | 0.567 |
| BMI (%) | | | 0.098 |
| Normal | 182 (70.3) | 78 (60.9) | |
| Obese | 5 (1.9) | 0 (0.0) | |
| Overweight | 56 (21.6) | 40 (31.2) | |
| Underweight | 15 (5.8) | 10 (7.8) | |
| Unknown | 1 (0.4) | 0 (0.0) | |
| PD-L1 ≥1% (%) | 178 (68.7) | 85 (66.4) | 0.731 |
| ECOG score ECOG=1 (%) | 185 (71.4) | 95 (74.2) | 0.648 |
| Smoking status (%) | | | 0.955 |
| Current | 46 (17.8) | 23 (18.0) | |
| Former | 122 (47.1) | 62 (48.4) | |
| Never | 91 (35.1) | 43 (33.6) | |

Abbreviations: BMI, body mass index; ECOG, Eastern Cooperative Oncology Group; PD-L1, programmed death-ligand 1;

[a] Patients treated with sintilimab combined with platinum-based chemotherapy.

**Supplemental Table 5. Comparison of different Multiple Instance Learning (MIL) models in TCGA-LUAD dataset.**

| | AUC of fold 1 | AUC of fold2 | AUC of fold 3 | Average AUC |
|---|---|---|---|---|
| BROW | 0.7699 | 0.7604 | 0.8128 | 0.7810 |
| PathoDuet | 0.7323 | 0.7357 | 0.7956 | 0.7545 |
| RetCCL | 0.7795 | 0.7645 | 0.8038 | 0.7826 |
| Phikon | 0.7764 | 0.7460 | 0.8265 | 0.7830 |
